# QI Project Promoting NP Compliance with an AOM Bundle in Pediatric Hospital-owned Retail Clinic

**DOI:** 10.1097/pq9.0000000000000537

**Published:** 2022-03-30

**Authors:** Kimberly R. Joo, Kelly Sandberg, Bonnie Albertini, Lisa Sowar, Lisa S. Ziemnik

**Affiliations:** From the 1 Urgent Care/Kids Express, Dayton Children’s Hospital, Dayton, Ohio; 2 Dayton Children’s Hospital, Dayton, Ohio.

## Abstract

The most common diagnosis for pediatric antibiotic prescriptions is acute otitis media (AOM). Therefore, it is logical to focus on AOM when striving to improve antibiotic prescribing in pediatrics. This quality improvement project aimed to improve documentation of diagnostic criteria and physical examination findings in the medical record and improve adherence to recommended antibiotic prescribing recommendations for AOM by nurse practitioners at a children’s hospital-owned pediatric retail clinic.

**Methods:**

We used The Institute for Healthcare Improvement’s Quality Improvement methodology to introduce an AOM quality bundle to providers in a pediatric retail clinic. We created Plan-Do-Study-Act ramps and implemented interventions, including NP education, electronic medical record improvements, and parent engagement. The percentage of compliant bundles (all five specific predetermined criteria successfully met) was measured for all patients diagnosed with AOM.

**Results:**

Baseline AOM bundle compliance began at a mean of 42%. Pareto analysis of baseline data indicated that antibiotic choice and duration were key failure modes. Antibiotic choice or duration errors occurred in 48% of reviewed charts at project inception. The interventions introduced throughout the project led to steady improvement in the percent of compliant bundles. The goal of 95% compliant bundles was achieved and maintained. At the project’s conclusion, 98% of antibiotic prescriptions were accurate.

**Conclusions:**

Implementation of multiple interventions with increasing levels of reliability improved the overall quality of documentation and increased the appropriate antibiotic prescriptions provided for patients diagnosed with AOM and seen by nurse practitioners at the retail clinic.

## INTRODUCTION

AOM is common in children. Approximately 80% of children are diagnosed with AOM by the age of 3 years.^1^ As a result, AOM is the most common reason that antibiotics are prescribed in pediatrics.^2^ Unfortunately, at least 30% of antibiotics prescribed in outpatient settings are unnecessary.^3^ This presents an opportunity for improvement by adhering to diagnostic criteria and antibiotic prescribing recommendations for AOM in this setting.^4^

An increasing number of pediatric patients are treated in outpatient retail clinics. The decision to focus a QI project on prescribers in this type of outpatient setting is imperative. Providers in this setting can promote responsible antibiotic prescribing by using the right antibiotic, dose, and duration. In addition, decreasing unnecessary antibiotic prescriptions can occur through uniform AOM diagnosis and management.^5^

The number of retail clinics in the United States has increased by over 10-fold to just under 2000 clinics in the past 10 years.^6,7^ Retail clinics offer a low-cost option for primary care. However there are limited data supporting the quality of care and patient satisfaction provided at retail clinics.^6,7^ Retail clinics are not new; however pediatric-hospital-owned retail clinics are an innovative alternative to adult-focused retail clinics. The American Academy of Pediatrics (AAP) and pediatricians have expressed concern over the possible effects on the quality of care for pediatric patients at adult-focused retail clinics.^6–8^ In one study, 37% of pediatric providers reported suboptimal care for their patients seen in adult outpatient retail clinics.^9^

Due to the number of unnecessary antibiotics prescribed in the outpatient setting and the AAP’s concern regarding pediatric patients seeking care in retail clinics, we decided to develop a QI project focused on the accurate diagnosis and treatment of AOM in our hospital-owned pediatric retail clinic. The specific aim for this improvement project was to increase the percentage of compliant AOM diagnostic and treatment bundles from 42% to 95% for pediatric patients seen in an outpatient pediatric hospital-owned retail clinic by July 1, 2020. We used the AAP guidelines for the diagnosis and management of AOM and the Center for Disease Control and Prevention (CDC) pediatric treatment recommendations for AOM to create five bundle criteria for this QI project.^2,10^

## METHODS

### Context

This project took place at a pediatric hospital-owned retail clinic located in a suburban setting in Ohio, with an approximate population of 8,000 children under the age of 21. The clinic opened on January 8, 2019, offering walk-in healthcare to meet the needs of children with low acuity episodic problems arising when primary care providers are not immediately available. This clinic solely provides acute care to its pediatric patients. Patients requiring chronic care management are referred to their primary care providers, who determine the need for further referral. Based on the clinical situation, all acute care needing advanced work-up is referred to either the pediatric urgent care or the pediatric emergency department. This pediatric retail clinic is staffed by pediatric-experienced nurse practitioners (NPs) and patient care assistants. The NPs have a collaborating physician available by phone during all operating hours. Patients either walk-in or reserve their place in line via the internet before arrival. The NPs and patient care assistants work together as a team to care for their pediatric patients and families.

The population included 1880 pediatric patients (from 6 weeks to 21 years) diagnosed by an NP with any type of AOM (as per ICD-10 coding) between the clinic opening day on January 6, 2019 and project completion on July 1, 2020. These 1880 patients accounted for 23% (1880/8060) of all patients seen at the retail clinic during the study period. Patients who presented with ear pain and did not receive a diagnosis of AOM were excluded from the project. NP staff expanded from four to fifteen throughout the project, due to strong clinic growth.

### Interventions

An application for project approval was completed and submitted to the Dayton Children’s Hospital Internal Review Board before implementation. The Internal Review Board determined this project was a QI project and thus exempt from full IRB review. The design and implementation of this project were organized using the Model for Improvement and Plan-Do-Study-Act (PDSA) Cycles.^11,12^

**Fig. 1.**
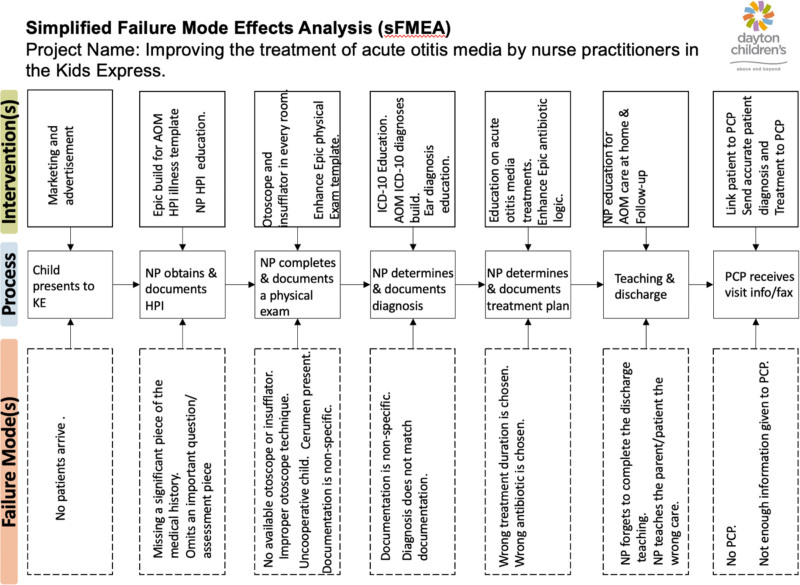
This simplified Failure Mode Effects Analysis outlines the process of patient care for a child with AOM, from registration through discharge at the pediatric retail clinic. It identifies possible failures and describes interventions to prevent failures from occurring.

The quality improvement (QI) team consisted of the medical director for the pediatric retail clinic (LZ) and grew from four to fifteen NPs by project conclusion. This team completed a simplified Failure Modes and Effects Analysis (sFMEA) before starting the project to determine processes for AOM assessment, diagnosis, and treatment and to identify possible process failures (Fig. 1).^11,12^ Identification of process failures led the team to develop a bundle of actions aimed at ensuring accurate assessment, diagnosis, documentation, and treatment of AOM. The team used the diagnosis and management guidelines from the AAP and CDC to identify qualifications for accurate AOM patient history, assessment, diagnosis, and treatment in children.^13^ Team members agreed on five consensus criteria that should be met to consider the patient’s assessment, diagnosis, and treatment as bundle compliant. This bundle included: (1) a history documented in the electronic medical record (EMR) that supports the diagnosis of AOM, (2) a documented physical examination performed by the provider that supports the diagnosis of AOM, (3) an AOM diagnosis with the corresponding ICD-10 code documented in the EMR, (4) NP documentation in the record justifying the medical decision to treat with an antibiotic in alignment with AAP & CDC guidelines, and (5) a prescription in the EMR, including the correct antibiotic choice, weight-based dose, and duration (Table 1).

**Table 1.** AOM Bundle Criteria

Bundle Element	Criteria Met	Criteria Not Met
**Patient history:** The patient history documented in the EMR supports the diagnosis of AOM.	The history documented in the EMR includes one of the following signs/symptoms: fever, ear pain, pulling ears, fussiness, crying, decreased appetite, not sleeping well	The history did not include at least one identified signs or symptoms of AOM.
**Physical examination:** The documented physical examination performed by the NP supports the diagnosis of AOM.	The physical examination performed and documented by the NP includes at least one of the following diagnostic criteria: erythematous TM, bulging TM	The physical examination performed and documented by the NP did not include any of the diagnostic criteria for AOM
**Diagnosis:** The AOM diagnosis has the correct ICD-10 code documented in the EMR.	The ICD-10 code documented by the NP in the EMR accurately reflects AOM.	The ICD-10 code documented by the NP in the EMR does not accurately reflect AOM.
**Medical decision-making:** The NP documentation supports the medical decision to treat with an antibiotic by following AAP & CDC guidelines.	The NP decision to treat the patient with antibiotics followed the current AAP & CDC guidelines and is documented in the EMR.	The NP decision to treat the patient with antibiotics did not follow the current AAP & CDC guidelines and/or is not documented in the EMR.
**Treatment:** The prescription in the EMR includes the correct antibiotic choice, dose, and duration.	The prescription in the EMR for an antibiotic includes the correct antibiotic choice, dose, and duration.	The prescription in the EMR for an antibiotic does not include the correct antibiotic choice, dose, and/or duration.

**Fig. 2.**
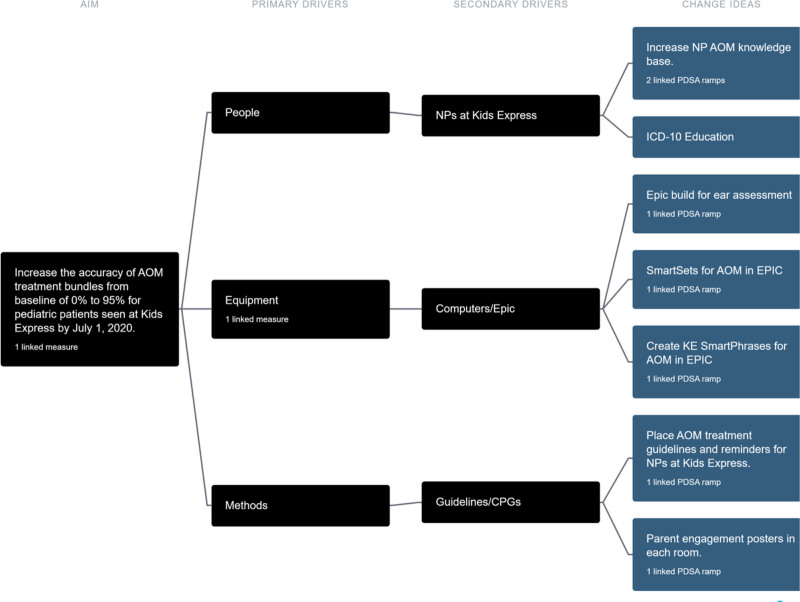
The Key Driver Diagram identified people, equipment, and methods as primary drivers for creating change for the care of AOM at the pediatric retail clinic. Multiple change ideas were linked to these through the secondary drivers of NPs, the EMR, and AOM guidelines.

The team developed a key driver diagram to depict a visual display (road map) of what they believed would contribute to the project’s success (Fig. 2).

### PDSA Ramps

**Fig. 3.**
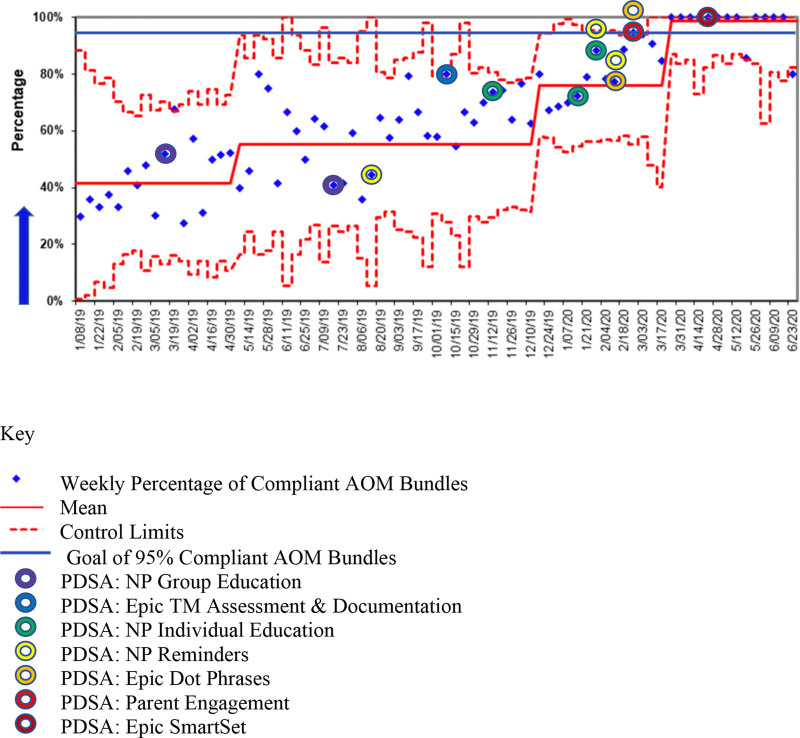
P-chart depicting the weekly percent compliance with AOM care bundle. The blue horizontal line represents the project goal of 95% bundle compliance. The colored rings denote a project intervention/PDSA ramp. Three centerline (mean) shifts in the percentage of compliant AOM bundles are noted.

This project included seven PDSA ramps, including 17 PDSA cycles from March 12, 2019 to May 27, 2020 (Fig. 3).

**Group Education:** The first PDSA ramp focused on AOM group NP education. This ramp began with an overview of assessment, diagnosis, and treatment for children with AOM before clinic opening. This information was reviewed in the form of a lecture and group discussion during a meeting, and then reinforced by an NP presentation during a subsequent education day.

**Epic TM Assessment & Documentation:** Chart audits revealed significant variability in tympanic membrane documentation among providers. In this second PDSA ramp, we updated the assessment documentation for TMs in the EMR to include a checklist of TM descriptive terms.

**NP Individual Education:** During this ramp, we changed education sessions to 1:1 with the QI team leader (KJ). Each NP received material outlining their errors in bundle criteria that required corrective action. The team leader reviewed the current status of the QI project, identified overall and individual areas that needed improvement in AOM care, and gave the NP an index card that summarized national antibiotic guidelines for AOM treatment.

**Fig. 4.**
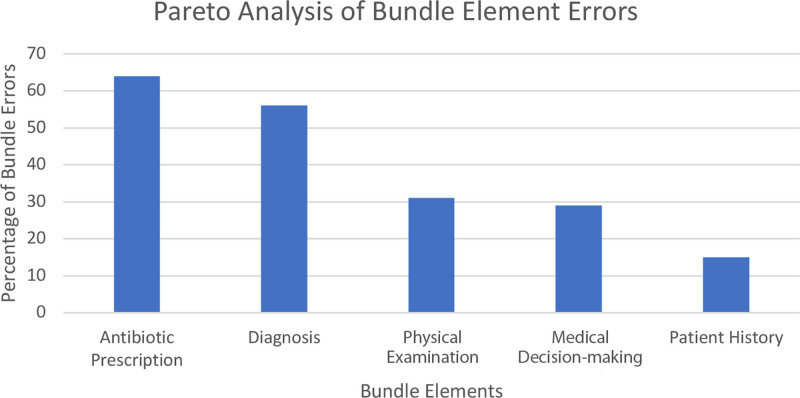
The Pareto chart identifies baseline bundle criteria failures during the first 8 weeks of the QI project. Antibiotic prescriptions and ICD-10 diagnosis criteria were the most common reasons for bundle non-compliance.

**NP Reminders:** This ramp included visual AOM antibiotic guideline reminders to improve the choice and duration of the prescribed antibiotic. Compliance with this intervention was identified by monitoring bundle adherence failures using a Pareto chart analysis (Fig. 4). The first cycle involved posting the current AOM treatment guidelines on a bulletin board at the clinic provider desk. However, the guidelines were lengthy and poorly utilized. Therefore, a smaller, more simplified version of the guideline reminder was laminated and attached to the computer screen in each examination room.

**Epic SmartPhrases:** In Epic, SmartPhrases or “dot phrases” are shortcuts for entering text quickly.^14^ SmartPhrases for several specific AOM treatments were written for use in the EMR. These phrases were categorized by patient’s age (<2 years or ≥2 years) and antibiotic prescribed (amoxicillin, amoxicillin/clavulanate, cefdinir, or ceftriaxone). Before placing these phrases in the EMR, all NPs reviewed the SmartPhrase changes. The phrases were available for use by all NPs at the clinic.

**Parent Engagement:** The goal of the sixth ramp was to improve communication with parents of children with AOM to educate them about guidelines for treating AOM. This education was an easy-to-read laminated poster, hung on the back of each examination room door. NPs discussion with parents of children with AOM included antibiotic choice and length of treatment.

**Epic SmartSet:** The creation of an AOM SmartSet was the final PDSA ramp in this project to increase the efficiency and accuracy of AOM treatment.^14^ The SmartSet for this project included AOM-specific diagnoses, discharge instructions, prescriptions, and charges. The SmartSet went live on April 23, 2020. We created an instructional PowerPoint using screenshots of Epic to assist with the adoption of this new tool. In addition, the QI team leader (KJ) met with each of the NPs 1:1 for personalized instruction.

The interventions in this project represented different reliability levels for healthcare. The interventions focused on education or training and reminders were lower reliability. Changes to EMR assessment documentation, the addition of SmartPhrases to the EMR, and the creation of a SmartSet for the EMR are higher level reliability interventions.^15,16^

### Measures

The data for this project were collected by the pediatric retail clinic medical director (LZ), by using an EMR generated list of patients with a diagnosis of otitis media and by manual chart review. Patients were identified by a report that searched all ICD-10 codes related to AOM and placed in an Excel file located on a secure, shared computer drive. Between January 8, 2019 and June 30, 2020, all medical charts for identified patients were reviewed weekly to assess compliance with each component of the AOM bundle. A bundle was compliant only if all five bundle element criteria were met. The team collected 77 weeks of data during the project. The total number of charts compliant with all bundle elements was divided by the total number of reviewed charts to obtain a percentage of compliant bundles per week. Weekly aggregated data were entered into LifeQI software, which generated germane statistical process control charts.^13^

### Analysis

A p-Chart was utilized to assist with data analysis (Fig. 3).^13,17^ AOM bundle compliance is depicted on the *y* axis, and time in weeks is represented on the *x* axis. Centerline shifts were justified based on standard statistical process control chart rules.^18^

The team created a Pareto chart during the first 8 weeks of this project to assist with identifying areas for improvement. Each time one of the five bundle criteria was not met; it was tabulated on the Pareto (Fig. 4). Non-compliant antibiotic prescription bundle elements were further analyzed to determine the leading cause of failure.

## RESULTS

The first eight weeks after clinic opening were used to determine baseline data. The percent compliance with the AOM bundle ranged from 30% to 50%, with a mean of 42% (Fig. 3).

During the project, three centerline shifts occurred in the percentage of compliant bundles (Fig. 3). After the individual NP education began, a moderate shift in the mean from 42% to 64% occurred. After the implementation of NP reminders and standardization of TM assessment documentation in Epic, a smaller shift upward in the mean occurred (64%–75%). The addition of Epic dot phrases and parent engagement resulted in a final centerline shift from 75% to 98%. The addition of EMR SmartSets assisted with maintaining the mean percent compliance above the project goal of 95%.

**Fig. 5.**
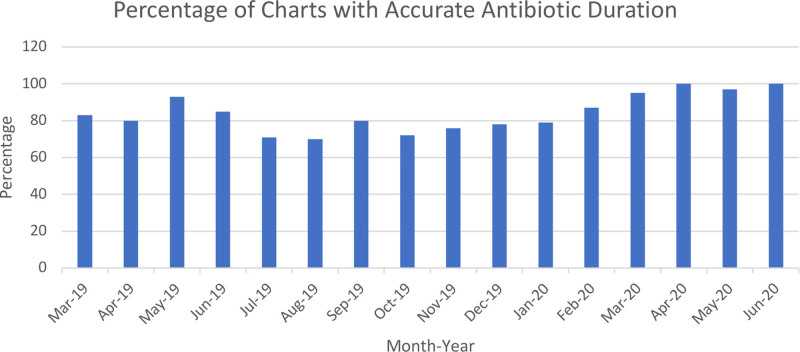
Antibiotic prescription issues were the most common bundle elements that were incorrect. This figure presents compliance with antibiotic duration recommendations by month.

The Pareto chart from the first 8 weeks of the project revealed a need for improvement in all five bundle criteria. Antibiotic prescription issues were the primary drivers of bundle non-compliance, followed by incorrect ICD-10 diagnosis (Fig. 4). Further analysis of the antibiotic prescription issues showed this was primarily due to incorrect prescribing of antibiotic duration. During the baseline evaluation, 45 of 184 antibiotic prescriptions were prescribed for an extra 3 days. This resulted in 135 unnecessary days of antibiotics. The compliance with prescribing the correct antibiotic duration improved toward the end of the project. During the last 10 weeks of the project, 98% of all patients treated for AOM were prescribed the correct antibiotic, at the correct weight-based dose, and duration (Fig. 5). Wrong antibiotic dose was not identified as a prescription error during the project.

## DISCUSSION

To our knowledge, this is the first QI project aimed at a specific pediatric diagnosis in the retail clinic setting. The low-level reliability interventions primarily focused on education resulted in positive improvements in AOM bundle compliance. Higher-level reliability interventions such as changes to the EMR, sustained and built upon these improvements over time. The project was successful with continued and sustained improvements over eighteen months, despite significant increases and decreases in the daily census, the addition of new NP staff, and the many changes that occurred due to the COVID pandemic in 2020.

The most significant improvements in AOM care bundle compliance occurred following NP education and NP reminders. Our findings are consistent with those of Weddle et al,^19^ who found that education improved antibiotic prescribing for NPs. We identified the antibiotic treatment bundle element as most in need of improvement multiple times during the project. Specific, individual errors pertaining to this bundle element were presented to the NPs during individual education sessions. Errors addressed during these sessions included choosing an inappropriate antibiotic or an incorrect duration of antibiotic treatment for AOM. Being aware of their individual mistakes was meaningful for the NPs. This self-awareness was a positive catalyst for change, and the NP reminders served as a visual reminder to support the change. The subsequent implementation of Epic SmartPhrases and SmartSets continued and sustained improvements after NP education, and visual reminders were in place.

This QI project was completed at one pediatric hospital-owned retail clinic; however, two more retail clinics were opened during the project timeline. These new clinics brought on additional NPs, bringing the core NP staff from four to fifteen. The addition of new NP staff during the project could have created a loss or slowing of improvement in bundle compliance due to a lack of knowledge of treatment guidelines, EMR SmartPhrases, and the EMR SmartSet. To account for those possibilities, careful consideration was made during the onboarding and orientation of each NP to educate them regarding the AOM treatment guidelines and EMR documentation.

The use of pneumatic otoscopy improves the accuracy of AOM diagnosis.^20^ Our NPs are all trained to perform pneumatic otoscopy and are educated about its importance; however, its routine use was not evaluated in this project. If the NP completed pneumatic otoscopy as part of the ear examination, it was included in the EMR. The decision not to require pneumatic otoscopy is a limitation of this QI project.

An additional provider did not confirm the diagnosis of AOM as part of this project because the clinic is staffed with only one NP. Accurate AOM diagnosis was presumed based on the education, training, and license of the NP and documentation of the physical examination. The clinic does not have its own patient base, nor does it have guaranteed follow-up or provider consistency. For this reason, wait-and-see and observation-only approaches may not have been utilized as often as they potentially could have been in another setting. This may have led to prescriptions for antibiotics that may not have been written if the patient had been seen in a primary care setting with consistent follow-up and an established patient relationship. These are all possible limitations to the project.

Another limitation of this QI project would be the lack of parent/patient satisfaction as a measurement of improvement. Future QI projects will consider creating a parent satisfaction survey for patients diagnosed with AOM to measure parent satisfaction with their AOM care. Overall parent satisfaction, using our hospital’s net promotor score rating of the clinic, remained high at over 95%.

At the beginning of the project, the daily patient census was low because the hospital-owned retail clinic was a new concept in our area for pediatric outpatient medical care. Patient volumes also fluctuated due to normal seasonal variation seen in pediatrics. At times, this afforded the NPs more time for the care of individual patients. During the first month, the clinic was open for business (January 2019), the NPs diagnosed approximately one to two patients per day with AOM, compared with six to seven patients per day in December 2019. The increase in census resulted in decreased time for the NP to spend with each patient. The clinic was designed for one NP to care for all patients and this did not change throughout the project. We were concerned that this increase in volume could have harmed the bundle compliance rate; however, this did play out, as bundle compliance remained stable or increased over time.

Another confounding factor that could have impacted this work was the COVID-19 pandemic in the Spring of 2020. The pandemic caused a drop in patient census from March through June of 2020, which forced the temporary closure of the two new clinics. The clinic hours were reduced from 10 to 8 hours a day during this time; however, it does not appear that these changes altered the course of improvement for this project.

This project’s sustainability is anticipated due to the multiple interventions with a higher level of reliability that were implemented toward the end of the project. The percentage of compliant bundles reached 100% for 12 of the last 14 weeks of the project.

## CONCLUDING SUMMARY

This QI project focused on optimizing the completion of AOM bundled diagnostic criteria aimed to: (1) improve accurate AOM history and physical examination finding’s documentation; (2) improve accurate ICD-10 AOM documentation for communication to the patient’s primary care provider; and (3) choose the correct option and duration of antibiotic treatment. The project produced consistent and sustained improvement in NP compliance with the AOM diagnostic bundle over time. We believe the implementation of multiple interventions with increasing levels of reliability to sustain improvements contributed to the project’s success. The interventions used in this project could easily be adopted by other pediatric retail clinics. We believe the principles underlying these interventions can be applied to other common pediatric diagnoses requiring antibiotics to foster responsible antibiotic prescribing habits in the pediatric or adult retail clinic setting. We plan to assess long-term sustainability of this work by continuing monthly monitoring for AOM bundle compliance to assess if this work might be applied to other common pediatric diagnoses seen in our clinic.

## DISCLOSURE

The authors have no financial interest to declare in relation to the content of this article.

## Acknowledgment

Special thanks to J. Michael Klatte, MD, Infectious Disease, Dayton Children’s Hospital, Dayton, OH, USA, for assistance with the editing of the early draft of this article.

Presented (as poster) at the Society for Pediatric Urgent Care 6th Annual Conference in virtual format, October 2020.

## References

[1] Atkinson H Wallis S Coatesworth AP. Acute otitis media. Postgrad Med. 2015;127:386–390. 25913598 10.1080/00325481.2015.1028872

[2] Centers for Disease Control and Prevention. Antibiotic prescribing and use in doctors’ offices: pediatric treatment recommendations. https://www.cdc.gov/antibiotic-use/community/for-hcp/outpatient-hcp/pediatric-treatment-rec.html. Published 2017. Accessed 11 November 2020.

[3] White AT Clark CM Sellick JA. Antibiotic stewardship targets in the outpatient setting. Am J Infect Control. 2019;47:858–863. 30862373 10.1016/j.ajic.2019.01.027

[4] Zetts RM Stoesz A Smith BA. Outpatient antibiotic use and the need for increased antibiotic stewardship efforts. Pediatrics. 2018;141:e20174124. 29793986 10.1542/peds.2017-4124

[5] Sanchez GV Fleming-Dutra KE Roberts RM. Core elements of outpatient antibiotic stewardship. MMWR Recomm Rep. 2016;65:1–12. 10.15585/mmwr.rr6506a1 27832047

[6] Hoff T Prout K. Comparing retail clinics with other sites of care: a systematic review of cost, quality, and patient satisfaction. Med Care. 2019;57:734–741. 31274781 10.1097/MLR.0000000000001164

[7] Duncan I Clark K Wang S. Cost and utilization of retail clinics vs. other providers for treatment of pediatric acute otitis media. Popul Health Manag. 2016;19:341–348. 26759922 10.1089/pop.2015.0051

[8] Garbutt JM Mandrell KM Sterkel R. Pediatric providers’ attitudes toward retail clinics. J Pediatr. 2013;163:1384.e6 1388.e6. 23810720 10.1016/j.jpeds.2013.05.008 PMC3812257

[9] Laughlin JJ Simon GR Baker C. AAP Principles concerning retail-based clinics. Policy statement. Pediatrics. 2006;118:2561–2562. https://pediatrics.aappublications.org/content/early/2014/02/18/peds.2013-4080 Accessed 11 November 2020. 17142546 10.1542/peds.2006-2681

[10] Lieberthal AS Carroll AE Chonmaitree T. The diagnosis and management of acute otitis media [published correction appears in Pediatrics. 2014 Feb;133(2):346. Dosage error in article text]. Pediatrics. 2013;131:e964 e999. 23439909 10.1542/peds.2012-3488

[11] Langley GL Moen RD Nolan KM. The Improvement Guide: A Practical Approach to Enhancing Organizational Performance. 2nd ed. Jossey-Bass; 2009.

[12] Scholtes PR Joiner BL Streibel BJ. The TEAM Handbook. 3rd ed. Goal QPC; 2018.

[13] LifeQI. Where people improve healthcare together. https://www.lifeqisystem.com/. Accessed 11 November 2020.

[14] Epic Systems Corporation (2017). Explanation of content tools in Epic. https://epictogetherny.org/Docs/Content_Tools_in_Epic.pdf. Accessed 7 December 2020.

[15] Toussaint J Mannon M. Improving reliability in healthcare. J Patient Saf. 2018;14:206–212. 26001554 10.1097/PTS.0000000000000195

[16] Nolan T Resar R Haraden C. Improving the reliability of health care. 2004. http://www.ihi.org/education/IHIOpenSchool/Courses/Documents/CourseraDocuments/08_ReliabilityWhitePaper2004revJune06.pdf. Accessed 11 November 2020.

[17] Duclos A Voirin N. The p-control chart: a tool for care improvement. Int J Qual Health Care. 2010;22:402–407. 20675711 10.1093/intqhc/mzq037

[18] Provost LP. The Health Care Data Guide: Learning from Data for Improvement. 1st ed. Wiley Imprint, U.S; 2011

[19] Weddle G Goldman J Myers A. Impact of an educational intervention to improve antibiotic prescribing for nurse practitioners in a pediatric urgent care center. J Pediatr Health Care. 2017;31:184–188. 27567148 10.1016/j.pedhc.2016.07.005

[20] Jones WS Kaleida PH. How helpful is pneumatic otoscopy in improving diagnostic accuracy? Pediatrics. 2003;112(3 Pt 1):510–513. 12949275 10.1542/peds.112.3.510

